# Supplementary material for: Dynamic transcriptomic profiles of zebrafish gills in response to zinc supplementation
Source: BMC Genomics. 2010 Oct 11;11:553. doi: 10.1186/1471-2164-11-553 (PMC3091702; doi:10.1186/1471-2164-11-553)
Supplement: Additional file 2 — Interactive Direct Interaction Network representing the molecular interactions between zinc, copper, iron, calcium and proteins encoded by transcripts changed by zinc supplementation. Mini web-site containing index.html and hyperlinked pages in subdirectory describing a Direct Interaction Network automatically generated based on curated interactions contained within the proprietary PathwayArchitect database. Ovals represent proteins and the circles symbolize metal ions. Objects are coloured by their abundance in zebrafish at the time-point they were significantly different from the control is a scale from -4 fold (dark green) to +4 fold (dark red). Where significant differences were found at more than one time-point, the colour overlay shows expression at the first instance. Dark blue squares denote 'binding', and light blue squares 'expression'; green squares stand for 'regulation', green diamonds for 'metabolism', and green circles for 'promoter binding'. Arrow heads indicate directionality of the interaction where annotated. All nodes and edges can be further interrogated by selecting the relative area of the image. [file 1471-2164-11-553-S2.zip › PathwayArchitect Zn xs DIN/748346.html]

# REGULATION:

|  |  |
| --- | --- |
| Type | REGULATION |
| Effect | Positive |


---

|  |  |
| --- | --- |
| Score | 0 |


---

|  |  |
| --- | --- |
| Reference Count | 8 |


---

|  |  |
| --- | --- |
| Mechanism | Unknown |


---

|  |  |
| --- | --- |
| Reference:0 || Sentence | "A threshold of iron concentration in the cell must be reached for the iron complex-induced increase of cellular Ca2+-uptake." |
| PMID | 9852309 |
| Year | 1998 |
| Journal | Int J Mol Med |
| RefScore | 1 |
| Source | PArchNLP |
  |
|  |  |


---

|  |  |
| --- | --- |
 Reference:1 || Sentence | "These results suggest that .OH formed by interaction of O2- and H2O2 in the presence of iron may play a major role in the HX-XO-induced disruption of airway epithelial Ca2+ homeostasis, and that NO potentiates ROS-induced [Ca2+]i response, presumably by reacting with O2- and producing .OH." |
| PMID | 10914332 |
| Year | 2000 |
| Journal | Exp Lung Res |
| RefScore | 2 |
| Source | PArchNLP |
  ||  |  |


---

|  |  |
| --- | --- |
 Reference:2 || Sentence | "Calcium channel blockers have no effects on the iron complex-cell interaction or iron-induced Ca2+-uptake modification." |
| PMID | 9852309 |
| Year | 1998 |
| Journal | Int J Mol Med |
| RefScore | 2 |
| Source | PArchNLP |
  ||  |  |


---

|  |  |
| --- | --- |
 Reference:3 || Sentence | "Iron is necessary for detectable MDA formation, but only Ca2+ and phosphate are necessary for the induction of membrane potential loss (Deltapsi) and Ca2+ release." |
| PMID | 12781777 |
| Year | 2003 |
| Species | Rat |
| Journal | Arch Biochem Biophys |
| RefScore | 1 |
| Source | PArchNLP |
  ||  |  |


---

|  |  |
| --- | --- |
 Reference:4 || Sentence | "The results showed that FP caused a marginal inhibition ( approximately 20%) of pump-mediated Ca(2+) extrusion and that non-heme iron induced a slight stimulation of the Ca(2+) efflux (11-20%), in contrast to the marked inhibitory effects on the Ca(2+)-Mg(2+)-ATPase of isolated membranes." |
| PMID | 10811972 |
| Year | 2000 |
| Species | Human |
| Journal | J Membr Biol |
| RefScore | 2 |
| Source | PArchNLP |
  ||  |  |


---

|  |  |
| --- | --- |
 Reference:5 || Sentence | "Accordingly, i) ALA undergoes transition metal-catalyzed oxidation to give O-2, H2O2 and HO.; ii) ALA induces iron release from ferritin, lipid peroxidation of cardiolipin-rich vesicles, single strand breaks in plasmid DNA, and guanosine oxidation in calf thymus DNA; iii) ALA causes Ca(2+)-mediated rat liver mitochondria permeabilization; iv) rats chronically treated with ALA exhibit increased glycolytic metabolism; v) brain extracts of ALA-treated rats reveal increased levels of thiobarbituric acid reactive substances, direct chemiluminescence intensity, carbonyl proteins, ferritin, and "free iron" and gamma-aminobutyric acid-receptor dissociation constant, and vi) patients with AIP and lead-exposed workers present augmented erythrocytic levels of the antioxidant enzymes superoxide dismutase and glutathione peroxidase." |
| PMID | 9070373 |
| Year | 1996 |
| Species | Human |
|  | Rat |
| Journal | Braz J Med Biol Res |
| RefScore | 1 |
| Source | PArchNLP |
  ||  |  |


---

|  |  |
| --- | --- |
 Reference:6 || Sentence | "This higher reactivity and the in vivo observed effect of increased brain uptake of iron from ferric lactate provoked by the presence of ATP, corroborate in vitro results showing an iron transfer from ferric lactate to ATP, as well as the mediator role of ATP in the iron-induced cellular Ca2+ homeostasis modification process." |
| PMID | 9142373 |
| Year | 1997 |
| Species | Mouse |
|  | Rat |
| Journal | Ann Clin Lab Sci |
| RefScore | 1 |
| Source | PArchNLP |
  ||  |  |


---

|  |  |
| --- | --- |
 Reference:7 || Sentence | "Iron-induced Ca2+ uptake was blocked by high concentrations of either desferrioxamine or methylprednisolone, whereas Ca2+ channel blockers did not affect Ca2+ uptake induced by Fe2+." |
| PMID | 2424624 |
| Year | 1985 |
| Species | Rat |
| Journal | Cent Nerv Syst Trauma |
| RefScore | 1 |
| Source | PArchNLP |
  |


---

|  |  |
| --- | --- |
